# Co-Delivery of Gemcitabine and Paclitaxel in cRGD-Modified Long Circulating Nanoparticles with Asymmetric Lipid Layers for Breast Cancer Treatment

**DOI:** 10.3390/molecules23112906

**Published:** 2018-11-07

**Authors:** Jing Zhang, Peng Zhang, Qian Zou, Xiang Li, Jianjiang Fu, Ying Luo, Xinli Liang, Yi Jin

**Affiliations:** 1 Key Laboratory of Modern Preparation of TCM, Ministry of Education, Jiangxi University of Traditional Chinese Medicine, Nanchang 330004, China; evens_zhang@163.com (J.Z.); yxmlinkzp1219@163.com (P.Z.); 18779118595@163.com (Q.Z.); jianjiang_fu@yeah.net (J.F.); luoyingkc@sina.com (Y.L.); xinlil@jxutcm.edu.cn (X.L.); 2 State Key Laboratory of Innovative Drug and Efficient Energy-Saving Pharmaceutical Equipment, Jiangxi University of Traditional Chinese Medicine, Nanchang 330006, China; 3 National Pharmaceutical Engineering Center for Solid Preparation in Chinese Herbal Medicine, Jiangxi University of Traditional Chinese Medicine, Nanchang 330006, China

**Keywords:** paclitaxel, gemcitabine monophosphate, cyclic RGD, pharmacokinetics, antitumor efficacy

## Abstract

Combination chemotherapy is a common clinical practice in cancer treatment. Here, cyclic RGD (arginylglycylaspartic acid) peptide was introduced to the surface of lipid/calcium/phosphate (LCP) asymmetric lipid layer nanoparticles for the co-delivery of paclitaxel (PTX) and gemcitabine monophosphate (GMP) (P/G-NPs). The sphere-like morphology of P/G-NPs displays a well-distributed particle size, and high entrapment efficiency and drug loading for both PTX and GMP, with a positive zeta potential. P/G-NPs were stable for up to 15 days. The cellular uptake of these cyclic RGD-modified nanoparticles was significantly higher than that of unmodified nanoparticles over 2 h incubation. Compared with the combination of free PTX and GMP (P/G-Free), P/G-NPs exhibited a longer circulation lifetime and improved absorption for PTX and GMP. Polyethylene glycol was responsible for a higher plasma concentration and a decreased apparent volume of distribution (V_z_). Nanoparticles enhanced the drug accumulation in tumors compared with other major organs after 24 h. P/G-NPs nearly halted tumor growth, with little evidence of general toxicity, whereas P/G-Free had only a modest inhibitory effect at 16 mg/kg of GMP and 2.0 mg/kg of PTX. Increased levels of apoptosis within tumors were detected in P/G-NPs group by approximately 43.6% (TUNEL assay). When compared with GMP NPs, PTX NPs, and P/G-Free, P/G-NPs decreased expression of B-cell lymphoma-2 and B-cell lymphoma-extra large proteins, and increased expression of cleaved poly-ADP-ribose polymerase-1. Calreticulin expression in tumors also increased upon the co-delivery of PTX and GMP. The antitumor effect of P/G-NPs is more powerful than P/G-Free, GMP NP, or PTX NP alone, without obvious toxicity.

## 1. Introduction

Breast cancer is the second most common cause of death from cancer and the most frequently diagnosed solid-organ cancer in women in the United States [1]. According to the Cancer Statistics, 2018, 268,670 new cancer cases and 41,400 cancer deaths (40,920 women, 480 men) are projected to occur in the United States at the end of 2018 [2]. Despite advances in the diagnosis of breast cancer, locally advanced breast cancer continues to be a major clinical problem, particularly in developing countries [3]. The incidence of this disease increases by 1–2% per year in developed countries, and by 5% per year in less developed countries [4]. The reasons for the different rates of increase among countries include timely diagnosis, level of education, economic status, screening and treatment programs, and so forth [5]. Therefore, there is an urgent need to enhance the efficacy of chemotherapy.

Taxanes are very effective anticancer drugs. The taxane paclitaxel (PTX) has long been used as the first-line therapy for breast cancer and ovarian cancer. Paclitaxel stabilizes microtubules and prevents the formation of normal mitotic apparatus, thereby blocking cancer cells at the G2/M phase. However, the therapeutic effectiveness of PTX is limited, due to its poor solubility in water, drug resistance, and side effects [6].

Gemcitabine (2′,2′-difluorodeoxycytidine (dFdC); GEM) is a pyrimidine nucleoside antimetabolite which has been approved for the treatment of non-small cell lung cancer and breast, bladder, and pancreatic cancer, as a chemotherapy agent. Its metabolites, dFdC-5′-monophosphate, dFdC-5′-diphosphate, and dFdC-5′-triphosphate, exhibit inhibition of several steps of nucleic acid metabolism [7]. The major mode of action lies in its triphosphate stopping division of cancer cells by incorporating its phosphorylated form at the end of the elongating DNA, and thereby preventing DNA polymerases from elongating [8]. Ribonucleotide reductase is also inhibited by dFdC-5′-diphosphate, which results in metabolic self-potentiating effects [7,9]. The therapeutic efficacy of gemcitabine monotherapy is limited, which is thought to arise from the rapid metabolism of GEM to 2′2′-difluorodeoxyuridine (dFdU), and which explains its short half-life (terminal t_1/2_ 8.0 min for 30 min infusions) [10]. Thus, GEM is often used in combination therapy for non-small cell lung cancer, ovarian cancer, and metastatic breast cancer [10,11,12].

A phase-III trial of a PTX and GEM (P/G) combination versus PTX alone showed that GEM added to PTX was an effective therapy for women with breast cancer who had received anthracyclines previously [13]. P/G combination chemotherapy is also one of the preferred regimens for patients with metastatic breast cancer. Combination chemotherapy of PTX and GEM was found to be an appropriate maintenance chemotherapy regimen, with survival benefits and a feasible toxicity profile in a large phase-III study conducted by the Korean Cancer Study Group [14]. It reduces the risk of intolerable side effects and maximizes the therapeutic effect, even at a lower dose [15,16,17].

However, co-delivery of GEM and PTX without carriers is often limited by several problems. For instance, GEM is a pro-drug, and its mechanism of action is based on intracellular phosphorylation into its active triphosphate and diphosphate derivatives. However, about ninety percent of GEM is eliminated rapidly and extensively in the blood, liver, and other tissues, mainly due to deamination to dFdU with little antitumor activity [18]. Paclitaxel is a highly lipophilic compound (log P ≈ 4 and solubility in water ≈ 0.4 μg/mL) [19], which hinders its ability to exert a pharmacodynamic effect. Furthermore, because of the different chemical and physical properties of GEM and PTX, the co-delivery of these two drugs is associated with substantial challenges. Moreover, the absence of pharmacokinetic interference between free GEM and free PTX has been reported [20]. Therefore, because of their different chemical and physical properties, free GEM and free PTX after intravenous administration is still distributed and eliminated independently. The different properties of GEM and PTX include the instability and low cellular permeability of GEM and the lipophilic nature of PTX. Therefore, different pharmacokinetics and biodistribution after co-administration of free GEM and free PTX could, consequently, lead to relapse/metastasis of tumors, due to low accumulation in cancer cells [21,22,23]. Therefore, nanocarriers were investigated to solve this problem of co-delivery of GEM and PTX into target cells.

The addition of the first phosphate group to form gemcitabine monophosphate (GMP, the bioactive form of GEM) is the rate-limiting step for GEM phosphorylation. We have “entrapped” GMP into lipid/calcium/phosphate (LCP) to realize significantly increased entrapment efficiency (EE%, the percentage of the drug that is entrapped into the nanoparticles) and decreased toxicity in myelosuppression [24,25,26]. To combine the therapeutic advantages of PTX and GEM, while also avoiding their delivery roadblocks, GMP instead of GEM can be encapsulated with PTX into LCP asymmetric lipid layer nanoparticles (P/G-NPs).

In addition, the modification of the targeting peptides on the nanoparticle surfaces represents an effective strategy for enhancing targeted distribution [27,28]. Cyclic RGD, sequence: cyclo(Arg–Gly–Asp–d-Phe–Cys), is a “tumor-homing” cyclic peptide which binds directly to αβ integrin (αvβ1, αvβ3, αvβ5, αvβ6, αvβ8, α5β1, α8β1, and αIIbβ3) [29]. Among these, integrin αvβ3 is important for the early stage of angiogenesis, which is overexpressed on the surface of cancer cells and tumor-vessel cells, but expressed at a low level in healthy vessel cells [30,31,32,33]. A drug delivery system grafted with cRGD peptides leads to receptor-mediated endocytosis [28]. The important receptors, α_v_β_3_ and Nrp1, are highly expressed in breast cancer. Therefore, cRGD induces more effective spread into extravascular tumor parenchyma, and modification of cRGD peptides on the surface of lipid–polymer NPs is an effective strategy [34,35].

We have previously developed nanoparticles that encapsulated GMP for treatment of non-small cell lung cancer and bladder cancer [24,25,26]. In both cases, high efficiency of drug entrapment was achieved with NP diameters of ≈45 nm for GMP NP. To the best of our knowledge, recent attempts to improve PTX/GMP delivery against cancer via nanosystems have been very limited. Herein, cRGD-modified LCP NPs were used to encapsulate PTX and GMP. The basic characteristics, pharmacokinetics, and pharmacological efficacy of these novel NPs were evaluated.

## 2. Results

### 2.1. Characterization of Nanoparticles

**Figure 1:**
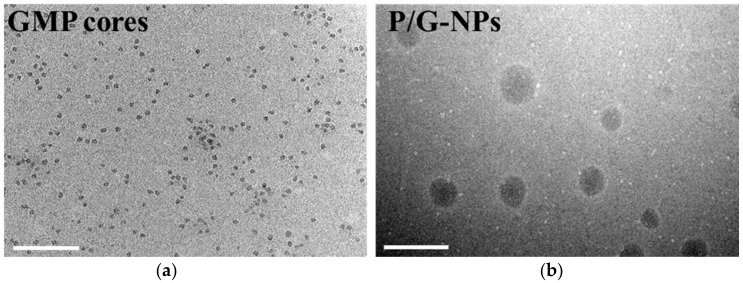
Morphological images: (**a**) gemcitabine monophosphate (GMP) cores; (**b**) lipid/calcium/phosphate asymmetric lipid layer nanoparticles for the co-delivery of PTX and GMP (P/G-NPs). Bar equals 200 nm.

In all cases, spherical or ellipsoid-shaped P/G-NPs were observed (Figure 1). The mean diameter of the P/G-NPs was 85.1 ± 8.1 nm. The zeta potential was 18.3 ± 0.63 mV, due to the cationic outer layer of 1,2-dioleoyl-3-trimethylammonium-propane chloride salt (DOTAP). The EE% of GMP and PTX was 93.6 ± 1.2% and 98.7 ± 0.5%, respectively. The DL% of GMP and PTX was 6.3 ± 0.1% and 0.8 ± 0.004%, respectively.

The stability of P/G-NPs was tested for 15 days at 4 °C (Table 1). The size of P/G-NPs did not exhibit a significant difference over 15 days (*p* < 0.5). The zeta potential and entrapment efficiency (EE%) showed slight changes from Day 1 to Day 15. These findings indicated that the P/G-NPs were stable over 15 days.

### 2.2. Cellular Uptake

**Figure 2:**
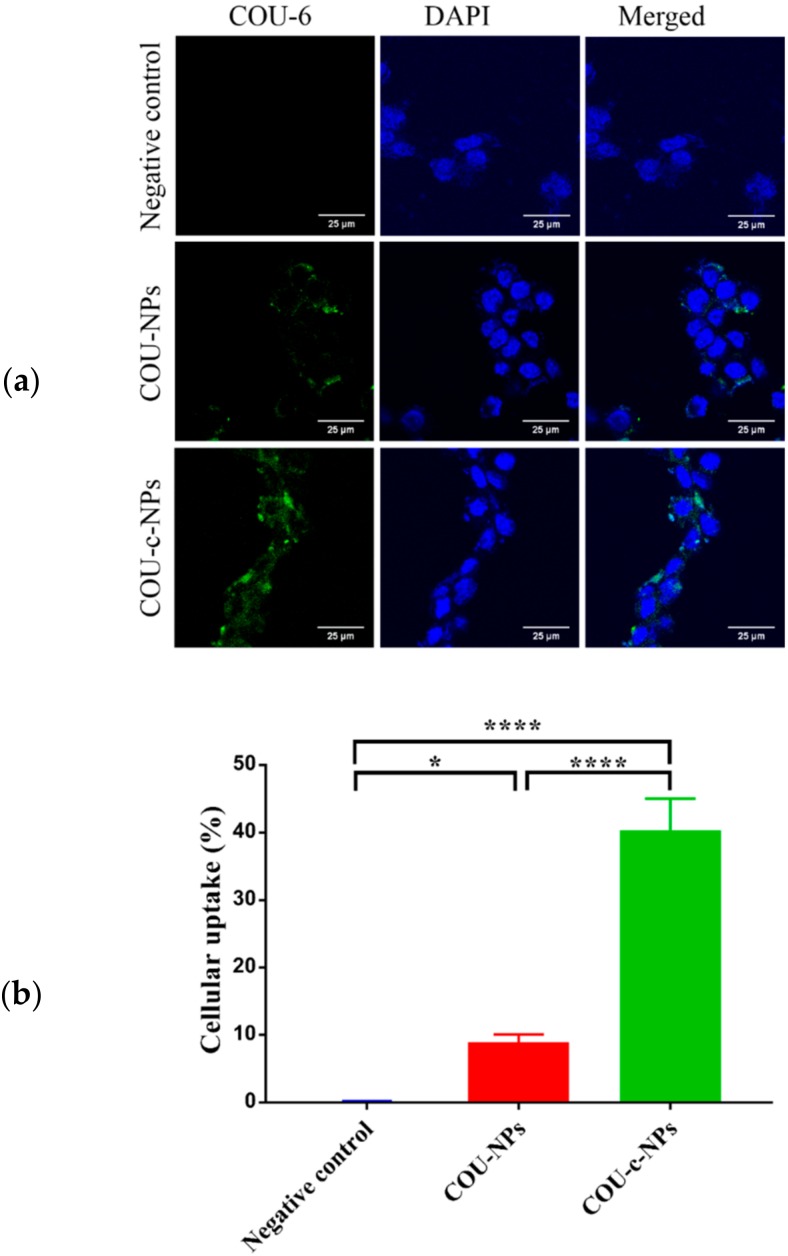
(**a**) In vitro CLSM images of MCF-7 cells treated with COU-NPs and COU-c-NPs for 2 h at 37 °C, 5% CO_2_. All pictures show the nuclei (blue), COU-6 (green), and merged images. Bar equals 25 μm; (**b**) Quantitative results of the cellular uptake of COU-6-loaded preparations expressed as the percentage of total cell number, **** *p* < 0.0001, * *p* < 0.05.

The internalization of cRGD-modified LCP asymmetric lipid layer nanoparticles was evaluated by using coumarin-6 (COU-6) as a fluorescence probe for Michigan Cancer Foundation-7 (MCF-7) cellular uptake by using confocal laser scanning microscopy (CLSM) [36]. The 1,2-distearoyl-*sn*-glycero-3-phosphoethanolamine-*N*-[methoxy(polyethylene glycol)-2000]-cRGD (DSPE-PEG2000-cRGD)-modified COU-6-loaded nanoparticles, COU-c-NPs, showed higher uptake than unmodified COU-loaded nanoparticles (COU-NPs) after 2 h incubation (Figure 2). Negligible fluorescence was observed in MCF-7 cells after incubating with phosphate buffered saline (PBS). Almost no autofluorescence was observed. The COU-c-NPs were endocytosed into cells. From these results, the targeting and elevated cellular uptake resulting from DSPE-PEG2000-cRGD modification was affirmed.

### 2.3. In Vitro Release

**Figure 3:**
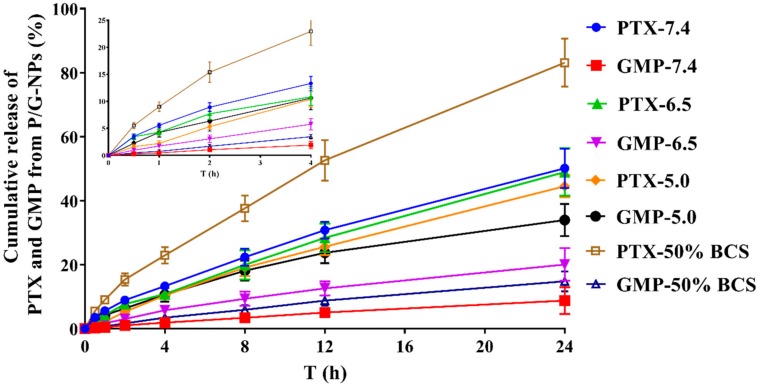
Cumulative release of PTX and GMP from P/G-NPs in different mediums (*n* = 3).

The cumulative release of GMP and PTX (%) in PBS containing 0.5% Tween 80 at different pH values, and PBS with 50% bovine calf serum (BCS) and 0.5% Tween 80 from P/G-NPs is shown in Figure 3. A pH of the release medium of 7.4, 6.5, and 5.0 was used to mimic the pH environment in blood, the tumor microenvironment, and lysosomes, respectively [37]. In P/G-NPs group, both PTX and GMP exhibited sustained release in this medium. P/G-NPs showed a non-significant burst release at the beginning, especially at pH 7.4 and 6.5. Paclitaxel exhibited higher release at pH 7.4, with a cumulative release of 50.1% in 24 h. The release of PTX was sustained in the medium at a lower pH value. Cumulative release of PTX over 24 h at pH 6.5 and 5.0 was 48.9% and 44.5%, respectively. When P/G-NPs were placed in a medium at pH 5.0, the release of GMP in P/G-NPs was 33.9% within 24 h, which was 3.8-fold and 1.7-fold greater compared with that at pH 7.4 and pH 6.5, in response to the acidic environment.

In order to mostly mimic blood conditions, we also used 50% BCS and 0.5% Tween 80 in PBS as the release medium, according to a previously published paper [38]. The release of PTX was faster in BCS-containing PBS, which was 1.8-fold greater than that in PBS at pH 7.4. The release of GMP exhibited a similar elevated cumulative release in BCS-containing medium.

### 2.4. Pharmacokinetics of P/G Formulations

**Figure 4:**
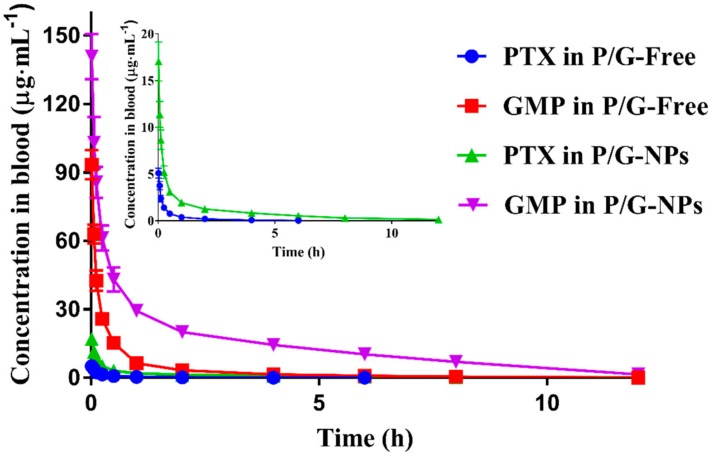
Blood concentration–time profiles of formulations after intravenous injection at 16 mg/kg of GMP and 2.0 mg/kg of PTX in rats (*n* = 6).

P/G-NPs exhibited prolonged elimination from the bloodstream, and a significantly higher maximum plasma concentration (C_max_) for both PTX and GMP, compared with P/G-Free (*p* < 0.05) (Figure 4 and Table 2), as calculated by PK solver software. The area under the concentration–time curve from zero to the final time point (AUC_0→t_) of PTX, calculated for the P/G-NP formulation, was 5.98-fold higher than that of the P/G-Free, with a 2.3-fold increment in the mean residence time from zero to the last time point (MRT_0→t_). The V_z_ for PTX and GMP in P/G-NPs was decreased significantly, by 50.3% and 72.1%, compared to that of P/G-Free, which suggested that the nanocarriers had a higher drug concentration in the blood, and slowed down drug distribution, compared with the free drug combination.

### 2.5. Tissue Distribution

**Figure 5:**
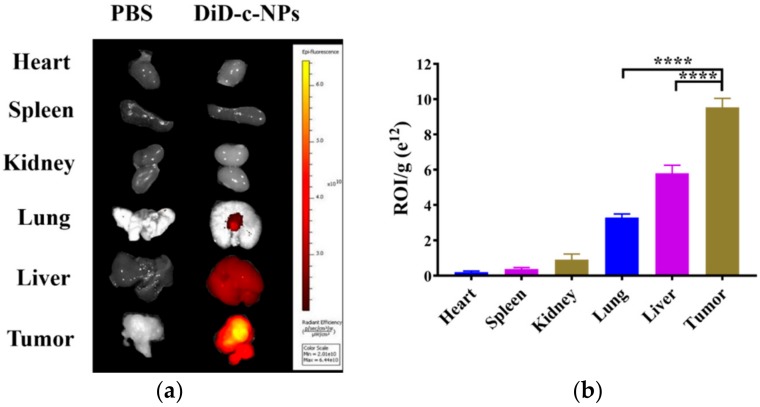
(**a**) Tissue distribution in tumor-bearing mice after injection DiD-c-NPs, and observed by in vivo imaging system, *n* = 3; (**b**) Region-of-interest (ROI) fluorescence intensities of tumors and major organs, **** *p* < 0.0001.

DiD (1,1′-dioctadecyl-3,3,3′,3′-tetramethylindodicarbocyanine, 4-chlorobenzenesulfonate salt) was used as the probe for nanoparticle distribution. DiD-loaded cRGD-modified NPs (DiD-c-NPs) were detected mainly distributed in the tumor after 24 h, in comparison with other organs (Figure 5a). Although an inevitable amount of nanoparticles accumulated in the liver or lungs, the amount was significantly lower than in the tumors (Figure 5b). With the help of nanoparticles with cRGD as a targeting ligand, most of the nanoparticles were taken up by tumor cells.

### 2.6. Antitumor Effect of P/G-NPs

**Figure 6:**
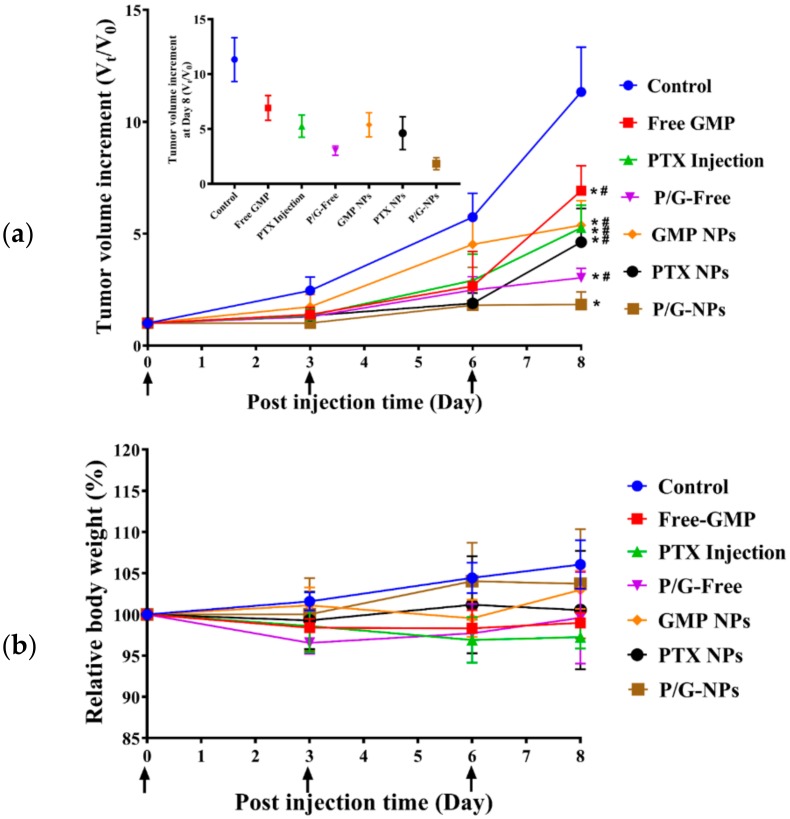
(**a**) Tumor growth inhibition effects of different formulations on MCF-7 tumor bearing mice. Free GMP, PTX Injection, P/G-Free, GMP NPs, PTX NPs, and P/G-NPs were administered intravenously every third day, for a total of three injections, as indicated by arrows. Data are mean ± SD. Statistics are as follows: * *p* < 0.05 vs Control; # *p* < 0.05 vs P/G-NPs; there is no significant difference among groups marked with “#”, *n* = 5; (**b**) The curve of relative body weight of every group (*n* = 5).

Monotherapy using Free GMP (16 mg/kg) and GMP NPs showed little antitumor effect, however, when combined with PTX, P/G-Free and P/G-NPs were more effective than GMP monotherapy (*p* < 0.05) (Figure 6a). GMP-NP-treated tumors had a significantly smaller volume than the six other groups at the end of the experiment, showing an increase in tumor volume of only 0.8-fold greater than at Day 1. Weight loss was not observed in any treatment group, suggesting that treatment was well tolerated (Figure 6b).

### 2.7. Induction of Significant Cell Apoptosis and Immunogenic Cell Death of P/G-NPs

**Figure 7:**
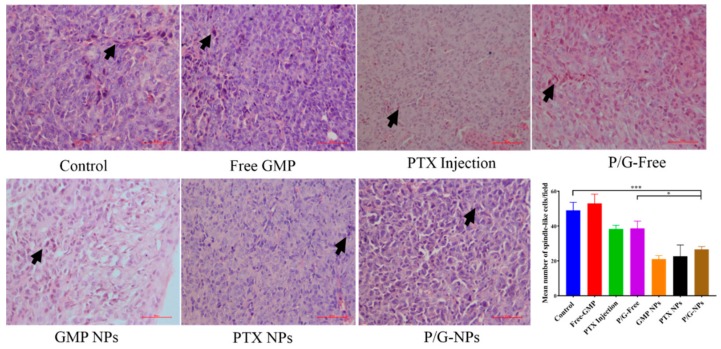
H&E stain of tumor tissue under different treatments, showing the presence of spindle-shaped cells (arrow) (200×), and the quantitative results of the mean number of spindle-like cells/field, *** *p* < 0.0005, * *p* < 0.05.

As shown by the hematoxylin and eosin (H&E) staining (Figure 7), the control tumor had many mitotic figures, showing the high mitotic activity of tumor cells [39]. Tumors that were treated with P/G-NPs experienced a dramatic decrease in the number of mitotic figures, and exhibited more basophilic and uniform nuclei. However, some chromosome condensation remained, due to the cytotoxicity induced by the combined therapy.

**Figure 8:**
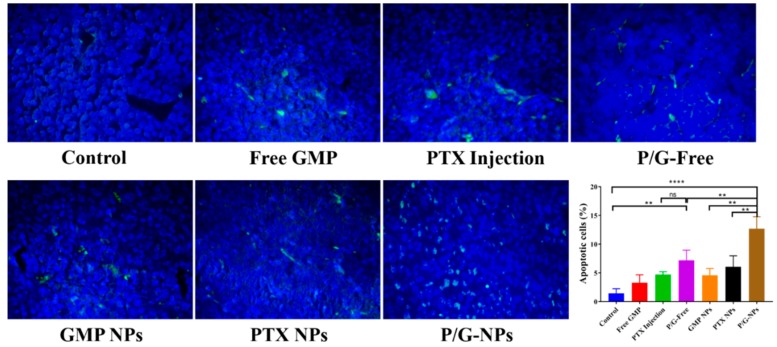
Effects of different treatments on the induction of apoptosis in tumors (400×), and the quantitative results of apoptotic cells expressed as the percentage of total cell numbers in tumors, **** *p* < 0.0001, ** *p* < 0.01, ns: not significant.

The TUNEL assay (Figure 8) revealed P/G-NPs to have the most effective killing effects, and to induce an 8.75-fold higher number of apoptotic cells compared with the control group. This effect was more potent than that of the P/G-Free treatment group, which showed 43.6% fewer apoptotic cells than the P/G-NPs. This finding is consistent with data in Figure 6, showing that the P/G-NPs exerted greater antitumor effects.

**Figure 9:**
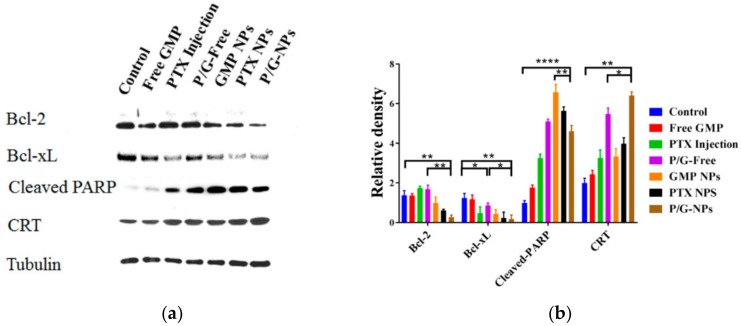
(**a**) Tumor lysates were prepared for Western blot analysis. (**b**) Relative densities of these markers in each group compared with tubulin control (*n* = 3), **** *p* < 0.0001, ** *p* < 0.01, * *p* < 0.05.

The Western blot analysis of tumor lysates is shown in Figure 9. GMP NPs, PTX NPs, and P/G-NPs caused significant reduction in the expression of Bcl-2 protein, especially for P/G-NPs, which caused an 80.1% reduction of expression compared with the control group (*p* < 0.01). We failed to observe a difference among Free GMP, PTX Injection, and P/G-Free groups in B-cell lymphoma-2 (Bcl-2) expression, when compared with the control group. However, when compared with the control group, the expression of B-cell lymphoma-extra large (Bcl-xL) was significantly decreased in both PTX Injection and P/G-Free. When compared with P/G-Free, P/G-NPs exhibited a significant decrement in the expression of Bcl-xL protein. We also observed significantly higher expression of cleaved poly-ADP-ribose polymerase-1 (Cleaved PARP) in the nanoparticle-treated groups. Besides the chemotoxic cell death caused by the combination of GMP and PTX, the increased calreticulin (CRT) expression in the P/G-NPs, which was 2.2-fold greater than that of the control group, also suggested the induction of immunogenic cell death (ICD). Compared with GMP NPs and PTX NPs, there was significantly increased expression of CRT in P/G-NPs, which was also increased by 17% over that of P/G-Free (*p* < 0.05).

### 2.8. Toxicity Analysis In Vivo

**Figure 10:**
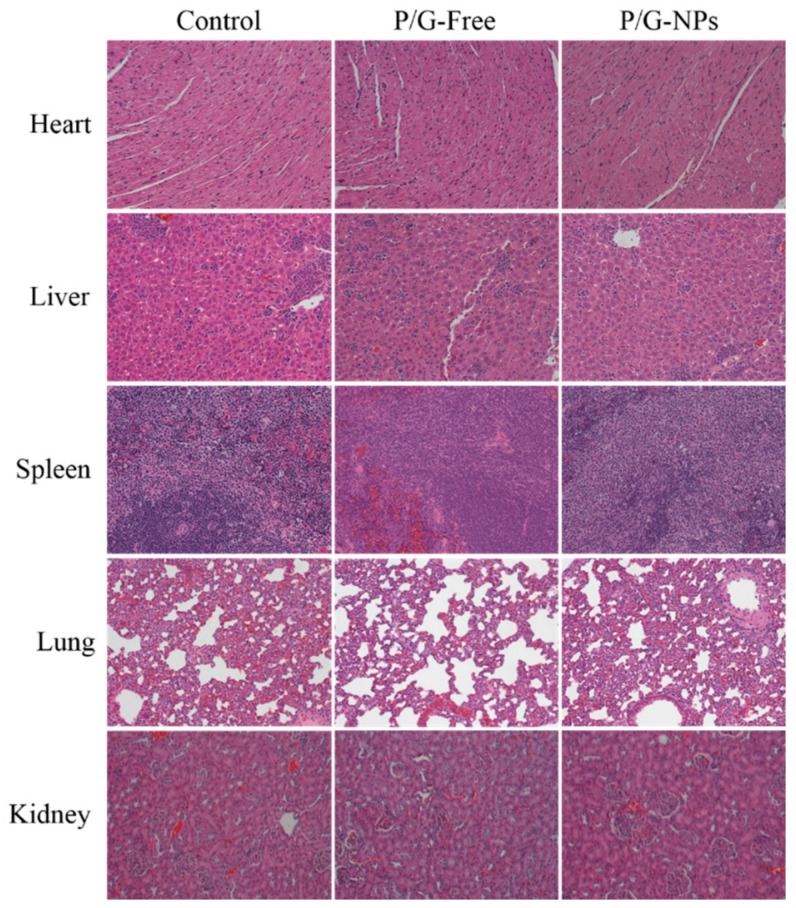
H&E staining of the PBS, P/G-Free, and P/G-NPs groups (20×).

**Figure 11:**
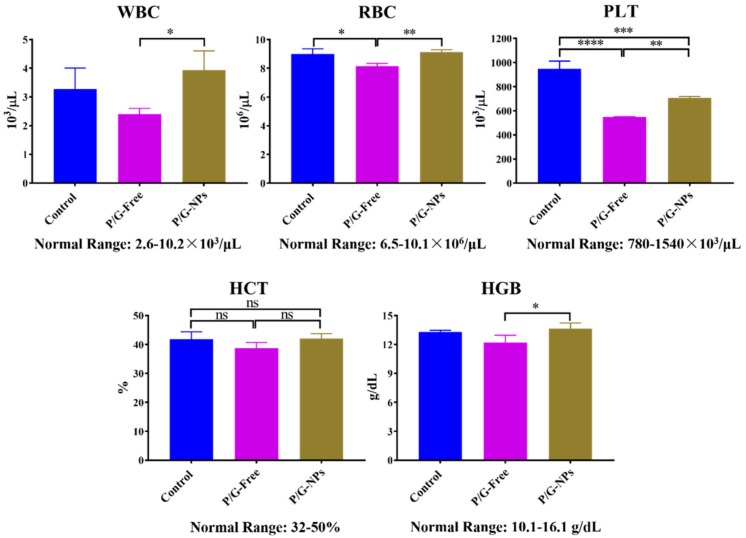
Hematology assay of the PBS, P/G-Free, and P/G-NPs groups (*n* = 3). **** *p* < 0.0001, *** *p* < 0.0002, ** *p* < 0.01, * *p* < 0.05, ns: not significant.

H&E staining of the PBS, P/G-Free, and P/G-NPs groups did not show obvious kidney injury, pulmonary toxicity, cardiac damage, or inflammatory infiltrates in the spleen (Figure 10). The combination of GMP and PTX did reduce the level of platelets (PLT) and red blood cells (RBC) compared to the untreated group (Figure 11). Blood tests showed that P/G-NPs could slightly increase the level of PLT, which was induced by chemotherapeutic drugs. There was no significant difference between P/G-NPs and the untreated group in the levels of white blood cells (WBC), RBC, hematocrit (HCT), and hemoglobin (HGB). Levels of alanine aminotransferase (ALT), aspartate aminotransferase (AST), lactate dehydrogenase (LDH) in all groups were in the normal range (Table 3). Serum levels of creatinine and blood urea nitrogen (BUN) were also in the normal range [24,25].

## 3. Discussion

Novel P/G-NPs were formulated, and the enhanced inhibition of tumor growth exhibited by P/G-NPs was evaluated. With regard to the combination of PTX and GMP, PTX stabilizes microtubules and prevents formation of normal mitotic apparatus, thereby blocking cancer cells at the G2/M phase [40]. Gemcitabine stops cancer-cell division [8,41]. The higher cumulative release of PTX over GMP in media demonstrated the sequential release of PTX over GMP, which may lead to a synergistic effect in vivo. The reason for the release profiles of PTX and GMP in different media was that PTX solubility decreased in media with a low pH, which resulted in a positive correlation between the rate of cumulative release and the pH of the medium. The interaction between the phosphate group of GMP and the calcium ions makes GMP become efficiently entrapped in the nanoparticle system containing calcium phosphate precipitate cores (lipid/calcium/phosphate (LCP) cores) [25]. The most important point is that calcium phosphate is an acid-sensitive material, which makes it dissolve rapidly in an acidic environment. Therefore, the GMP cores were designed to release GMP into the cytoplasm in the acidic lysosomal or endosomal environment [42,43]. The GMP cores are comprised of calcium phosphate, therefore, GMP release at pH 7.4 was slow, and then increased rapidly under acidic conditions, which made the cumulative release rate of GMP inversely correlated with the pH of the medium. Due to the effect of phospholipase in BCS-containing PBS, PTX loaded in the layer of lecithin of the nanoparticles was released significantly faster than that in PBS.

It has been demonstrated that the synergistic effect of the PTX/GEM combination is ratio- and regimen-dependent. The synergistic effect is more pronounced if PTX is administered before GEM [5], which was confirmed by the drug-release behavior observed. The importance of sequential dosing may be because PTX induces inactivation of the enzymes that can destroy GEM [44]. The better and faster release behavior of PTX over GMP in P/G-NPs may contribute to the antitumor effect. However, because the lipid membrane has the release characteristics of membrane fusion after contact with cells in vivo, in vitro drug release cannot completely simulate actual drug-release behavior in vivo [45,46].

The antitumor effect of P/G-NPs could be attributed to the endocytosis mediated by the “size-dependent” enhanced permeability and retention effect. This is caused by tumor vasculature, which is comprised of poorly aligned and defective endothelial cells lacking innervation, as affirmed by their pharmacokinetic behavior (Table 2). Secondly, cRGD peptides were grafted onto 1,2-distearoyl-*sn*-glycero-3-phosphoethanolamine-*N*-[methoxy(polyethylene glycol)-2000] (DSPE-PEG2000) to obtain tumor targeting, and were inserted in the NP surface. cRGD peptide is frequently utilized to improve the tumor penetration effects of nanocarriers [34,35,36].

In breast cancers, the Bcl-2 protein family is essential for development and homeostasis. Sustained Bcl-2 family dysregulation is a hallmark of cancer. Bcl-2 and Bcl-xL, in the Bcl-2 protein family, inhibit both proliferation and apoptosis [47]. Bcl-2 expression has been shown to be a favorable prognostic factor and predictor of the response to endocrine therapy [48]. It has been reported that tamoxifen can induce apoptosis in a time- and dose-dependent manner by modulating Bcl-2 levels in MCF-7 breast cancer cells, which are characterized by overexpression of human epidermal growth factor receptor 2 [49]. Here, we assumed that P/G-NPs could act like tamoxifen to modulate Bcl-2 levels in breast cancer cells, and lead to cell apoptosis. It was reported that there are both quantitative and qualitative differences in the functional activity of Bcl-xL and Bcl-2 in different cells. Bcl-xL is approximately 10 times more active than Bcl-2 in MCF-7 cells in repressing apoptosis induced by chemotherapy drugs [50]. The nanocarriers for GMP and/or PTX could significantly decrease the expression of Bcl-xL. Meanwhile, cleavage of PARP by caspases is considered to be a hallmark of apoptosis [51]. GMP entrapped in the combined P/G-Free, P/G-NPs, and GMP NPs was shown to inhibit the pro-survival program, as evidenced by increased PARP cleavage (Figure 8), which led to significant induction of cell apoptosis in tumors (Figure 7).

Various clinical trials have studied the associations between tumors and the host immune responses induced by immunotherapy. CRT is a luminal calcium-binding protein in the endoplasmic reticulum [52], which is the general marker for mammalian apoptotic cells to be recognized by phagocytes, and for the translocation of CRT from the endoplasmic reticulum to the cell surface, which acts as an “eat me” signal for dendritic cells [53]. CRT overexpression has been reported to contribute to the diminution of cell viability when compared with its control in MCF-7 cells [54]. Recent studies have shown that low-dose PTX can improve the effect of cytokine immunotherapy by modulating the immunosuppressive tumor microenvironment, including the cytokine network and inhibitory activity of regulatory T cells [55]. Moreover, GEM has been reported to improve the immunogenicity of cancer through CRT translocation in CT-26, B16, and Lewis lung carcinoma cell lines [56]. Therefore, we hypothesized that the increased number of apoptotic cells in tumors could contribute (at least in part) to the immunogenicity caused by the chemotherapy of a combination of PTX and GMP. Further study of the ICD-inducing effect of combination NPs is ongoing. The antitumor activity of P/G-NPs may result from increased uptake of chemotherapeutic drugs by the tumor, reduced toxicity, apoptosis of cancer cells, and ICD induction by co-delivery of PTX and GMP.

Besides the combinatorial effects, safety is also crucial for the chemotherapy. The systemic toxicity of various formulations was evaluated in vivo by analyses of serum chemistry, hematology assay, and histology of major organs. These studies showed that the PTX and GMP combination was non-toxic to major organs and tissues, and did not cause obvious toxicity.

## 4. Materials and Methods

### 4.1. Materials

PTX (purity ≥ 97%) was purchased from Sigma-Aldrich (St. Louis, MO, USA). The PTX Injection was obtained from Sichuan Sunnyhope Pharmaceutical Co., Ltd. (Batch number 1604201; China Food and Drug Administration (CFDA) approval number H20046119; Sichuan, China). The internal standards docetaxel (purity ≥ 99.0%) and cefaclor (purity = 94.4%) were purchased from Shanghai Tauto Biotechnology Co., Ltd. (Shanghai, China) and the National Institute for Food and Drug Control (Beijing, China). DSPE-PEG2000 and highly purified cholesterol were obtained from Shanghai Advanced Vehicle Technology Pharmaceutical Ltd. (Shanghai, China). The DSPE-PEG2000-cRGD was from Xi’an Ruixi Biological Technology Co., Ltd. (Xi’an, China). DOTAP and dioleoyl phosphatidic acid (DOPA) were purchased from Avanti Polar Lipids (Alabaster, AL, USA). DiD’ solid (1,1′-dioctadecyl-3,3,3′,3′-tetramethylindodicarbocyanine, 4-chlorobenzenesulfonate salt) was obtained from Invitrogen (Carlsbad, CA, USA). COU-6 was purchased from Sigma-Aldrich (St. Louis, MO, USA). DeadEnd^TM^ Fluorometric TUNEL assay kits were obtained from Promega (Madison, WI, USA). Antifade Mounting Medium with DAPI (4′,6-diamidino-2-phenylindole) was obtained from Vector Laboratories (Burlingame, CA, USA), and BCS was obtained from Gibco (Carlsbad, CA, USA). All other chemicals were of analytical grade and were used as received.

The human breast adenocarcinoma cell line MCF-7 was obtained from Beijing Dingguo Changsheng Biotechnology (Beijing, China). The MCF-7 cells were cultured in RPMI 1640 (Life Technologies, Carlsbad, CA, USA) containing 10% fetal bovine serum (Life Technologies), L-glutamine (2 mM), and penicillin–streptomycin solution (40 U/mL-each; Life Technologies). Cells were cultivated in a humidified incubator at 37 °C and 5% CO_2_. Cells were harvested with 0.05% trypsin-EDTA before subculture.

### 4.2. Animals

The animal experiments were carried out in accordance with the Guide for the Care and Use of Laboratory Animals (National Research Council of China, Beijing, China). The study protocol (JXUTCM 2015013, 08/19/2015) was approved by the Institutional Animal Care and Use Committee of Jiangxi University of Traditional Chinese Medicine (Nanchang, China).

Female athymic nude mice (6–8 weeks; 22–28 g) and male Sprague-Dawley rats (200 ± 20 g) were provided by Hunan SJA Laboratory Animals (Hunan, China). The animals were cared for in the animal experimental center at Jiangxi University of Traditional Chinese Medicine. The animal room was well ventilated and had a regular 12 h light–dark cycle throughout the experimental period.

### 4.3. Preparation of DOPA-Coated GMP Cores

DOPA-coated GMP cores were prepared according to our previous studies with some adjustments [16,17]. Briefly, 600 μL of 2.5 M CaCl_2_ was added to 20 mL of oil phase, composed of Igepal CO-520/cyclohexane (29/71, *v*/*v*). The other emulsion was prepared by adding 150 μL of 50 mM Na_2_HPO_4_, 180 μL of 60 mM GMP solution, and 270 μL H_2_O into the same oil phase. Four hundred mL of 20 mM DOPA was added to the phosphate phase, then the two separate emulsions were mixed for 20 min. Another 400 μL of 20 mM DOPA was added to the mixture before adding 40 mL absolute ethanol. The ethanol mixture was centrifuged at 10,000 *g* for 15 min to pellet the GMP cores and remove the surfactants and cyclohexane. The GMP cores were washed twice with absolute ethanol and dried under N_2_. The GMP cores were then redispersed in chloroform, and stored in a glass vial for further use.

### 4.4. Preparation of P/G-NPs Loads with Different Cargos

To prepare the final cRGD-functionalized P/G-NPs with an outer lipid layer, 2 mL DOPA-coated GMP cores in chloroform containing 2.8 mM GMP were mixed with 420 μL of 0.85 mM PTX, 250 μL of 25.8 mM cholesterol, 170 μL of 35.8 mM DOTAP, 510 μL of 8.9 mM DSPE-PEG2000, and 57 μL of 7.4 mM DSPE-PEG-cRGD. Then, the organic solvent was removed using a rotary vacuum evaporator, and it was dried further under a constant flow of nitrogen for 30 min to form a drug-containing lipid membrane. Residual lipids were dissolved in 200 μL of tetrahydrofuran followed by 90 μL of absolute ethanol, and then suspended in 1.8 mL of water. The suspension was then extruded through a membrane (10 mL LIPEX™ Thermobarrel Extruder with Whatman PC MB 25 mm 0.08 μm membrane LOT#:138360) with 80 nm diameter pores under 300 psi pressure generated by N_2_ gas. The obtained suspension was dialyzed in distilled water for 120 min to remove the THF and ethanol. The obtained suspension was filtered through a 0.22 μm filter to remove bacteria, and stored at 4 °C. The preparation procedure of PTX NPs was identical to that of P/G-NPs, except that GMP cores were replaced with empty cores. Simultaneously, GMP NPs were prepared in an identical manner to the P/G-NPs, except that PTX solution was not included in the lipid solution.

For the cellular uptake experiment, COU-6 was employed as fluorescence probe. The prepare protocol of COU-6-loaded cRGD-modified NPs (COU-c-NPs) was identical to that of PTX NPs, except that PTX was replaced by COU-6 at a concentration of 100 μg/mL. The formulation of COU-6-loaded NPs without DSPE-PEG2000-cRGD (COU-NPs) was the same of that of COU-c-NPs, except that DSPE-PEG2000-cRGD was replaced by DSPE-PEG2000.

For the biodistribution assay, DiD was employed as a fluorescence probe. The preparation protocol of DiD-loaded cRGD-modified NPs (DiD-c-NPs) was identical to that of PTX NPs, except that PTX was replaced by DiD at a concentration of 50 μg/mL.

### 4.5. Characterization of P/G-NPs

The P/G-NPs were viewed under an electron microscope (JEM-1200 EX; JEOL, Akishima, Japan) by conventional negative staining methods using a 0.3% phosphotungstic acid buffer (pH 7.0). The size and zeta potential of new nanoparticle formulations over time were evaluated using a Nano ZS90 apparatus (Malvern Instruments, Malvern, UK). The surface electric charge of nanoparticles at 25 °C was determined. The dispersion medium was filtered (Millipore; Bedford, MA, USA) water (pH 7.0), and ions were eliminated to stop them influencing the surface electric charge of the nanoparticles.

The entrapment efficiency (EE) of GMP and PTX were determined using an ultrafiltration method for separating the non-entrapped drug from NPs [47]. Briefly, 500 μL of drug-loaded P/G-NPs were placed in an ultrafiltration tube (Nanosep MF; Pall Corporation, Port Washington, NY, USA) fitted with a filter membrane (molecular weight cutoff: 10 kDa). Free drugs in the underlying solution were collected by centrifugation at 8000 rpm for 15 min using a high-speed refrigerated centrifuge (3–18 K; SIGMA Laborzentrifugen GmbH, Osterode, Germany). The drug content of GMP and PTX in the ultrafiltrate (c_free_) was determined simultaneously by high-performance liquid chromatography (HPLC) on a ZORBAX SB-C18 column (250 mm × 4.6 mm, 5 μm; Agilent Technologies, Santa Clara, CA, USA) at 268 nm. The mobile phase was 0.06 M ammonium acetate solution (pH 5.7, adjusted using glacial acetic acid)-acetonitrile. A gradient elution was used with a flow rate of 1.0 mL/min, where initially 5% organic solvents (acetonitrile containing ammonium acetate solution) were held for 7 min, then increased linearly to 70% over 10 min, where they was held for another 8 min and, finally, decreased linearly to 5% over 10 min, where they were held until the end of a 5 min run. The column temperature was maintained at 25 °C, and the injection volume was 10 μL. Then, 0.5 mL of suspension was diluted with 2.0 mL of a mixture of tetrahydrofuran and 1 M HCl (70/30 *v*/*v*) solution, to determine the total drug concentration (*c_total_*) by HPLC. The EE% and drug loading (DL%, wt %) were calculated using the following Equations (1) and (2):

(1) $$\mathrm{EE}\%=\frac{C_{total}-C_{free}}{C_{total}}\times 100,$$

(2) $$\mathrm{DL}\%=\frac{C_{total}-C_{free}}{Mass\;of\;NPs}\times 100$$.

In addition, the change in particle size, zeta potential, and EE% for 15 days during storage at 4 °C was carried out for the stability evaluation.

### 4.6. Cellular Uptake

MCF-7 cells were seeded in 35 mm cell culture dishes with glass bottoms at a density of 2 × 10^5^ per dish, and incubated for 24 h before treatment. COU-c-NPs and COU-NPs were added to the dishes at the same concentration, and the cells were cultured for another 2 h at 37 °C, 5% CO_2_ condition. Ice-cold PBS was used to remove the absorptive and free particles three times, and then the cells were fixed by 4% paraformaldehyde. After fixation, DAPI Mounting Medium was added for nuclear staining. The cellular uptake of the NPs was observed using a LSM 710 confocal microscope from Zeiss (Zeiss, Oberkochen, Germany) and quantified by Image J software (National Institute of Health).

### 4.7. In Vitro Drug Release

Analyses of drug release in vitro were undertaken using a Modified US Pharmacopoeia Apparatus 4 with flow-through cells of 12 mm diameter (CE7 Smart; Sotax, Horsham, PA, USA) packed with glass beads (1 mm) in closed-system mode at 37 °C [57]. PTX and GMP were simultaneously analyzed by the HPLC system mentioned before. The preparations were dispersed in cells using 100 mL of dissolution medium at a flow rate of 4 mL/min. The release media were phosphate-buffered saline (PBS) containing 0.5% Tween 80 at pH 7.4, 6.5, and 5.0, because of the low solubility of PTX in water, and PBS containing 50% BCS and 0.5% Tween 80 to mimic blood conditions [38]. Samples (1 mL) were withdrawn and replenished with an equal volume of blank fresh media. For evaluation of release in saline, samples from PBS media were analyzed directly by HPLC, as mentioned above. Samples of the mimic blood (50% BCS and 0.5% Tween 80 in PBS) were processed and analyzed directly by UHPLC/mass spectrometry as mentioned in Section 4.8. Media replenishment was taken into account for calculation of the cumulative percent release. All measurements were conducted in triplicate, and the mean values and standard deviations are reported.

### 4.8. Pharmacokinetics Studies

Twelve healthy Sprague-Dawley rats (200 ± 20 g) were divided randomly into two groups and fasted overnight before experimentation. Rats in these two groups were injected (iv) into the tail vein with a mixed solution of GMP and PTX (P/G-Free) and P/G-NPs, at 16 mg/kg for GMP and 2 mg/kg for PTX. After injection at designated times (0.0167, 0.066, 0.12, 0.25, 0.5, 1, 2, 4, 6, 8, and 12 h), blood samples (500 μL) were withdrawn from the retro-orbital plexus. The blood samples were centrifuged at 6000 rpm for 5 min at room temperature, and 200 μL of the separated plasma maintained at −80 °C for analysis.

One hundred microliters of the internal standards docetaxel (1.0 μg/mL) and cefaclor (0.1 μg/mL) were added to the samples to determine PTX and GMP, respectively. A working solution was added to the sample before addition of methanol (300 μL) to precipitate proteins. The mixture was centrifuged at 6000 rpm for 10 min at room temperature to dissolve the drug in the organic solvent. The obtained supernatant was subjected to UHPLC/mass spectrometry for the detection of GMP and PTX using a TRIPLE QUDA 4500 liquid chromatograph triple quadrupole mass spectrometer equipped with an electrospray ion source in positive mode (AB SCIEX, Framingham, MA, USA).

Chromatographic separation was determined on a XB-C18 Ultimate UHPLC column (21 mm × 50 mm, 1.8 µm, Welch Materials, TX, USA). Gradient elution was undertaken using solvent A (0.1% formic acid solution) and solvent B (acetonitrile). Gradient elution was completed at a flow rate of 0.28 mL/min. Initially, 50% organic solvent (acetonitrile containing formic acid solution) was used from 0.01 min to 1 min, and increased linearly to 55% in 0.5 min, where it was held for another 1 min, and then increased to 96% in another 0.5 min and, finally, decreased to 10% at 4.6 min, where it was held until the end of the 2 min run.

The mass spectrometer operated in positive ion mode within multi-ion reaction monitoring mode. The ion-reaction ratios for the quantitative analyses of GMP and the internal standard cefaclor were *m*/*z* 342.0 → *m*/*z* 301.2, and *m*/*z* 368.1 → *m*/*z* 174.1, respectively. The collision energy of GMP and the internal standard was 15 V and 16 V, respectively. The ion-reaction ratios for the quantitative analyses of PTX and the internal standard docetaxel were *m*/*z* 876.3 → *m*/*z* 308.1, and *m*/*z* 830.5 → *m*/*z* 549.3, respectively. The collision energy for PTX and the internal standard was 37 V and 36 V, respectively. Ionization conditions included the use of an electrospray ion source with an injection voltage of 5.5 kV, an ion source temperature of 600 °C, 55 psi for GS1 and GS2 pressures, and 7 psi for the collision gas pressure.

The area under the concentration–time curve from zero to the final time point (AUC_0→t_), the mean residence time from zero to the last time point (MRT_0 → t_), the total plasma clearance (CL), the apparent volume of distribution (V_z_), and maximum plasma concentration (C_max_) of the drug, were obtained.

### 4.9. Antitumor Efficacy In Vivo

Primarily, BALB/c female nude mice were used to create the breast-tumor model by tumor tissue transplantation. MCF-7 cells (1 × 10^7^ per mouse) were injected into the mammary gland via subcutaneous inoculation. When the tumor volume reached ≈1000 mm^3^, the tumor was collected and cut into pieces with a diameter of 1.5 mm. Then, these small tumor pieces were transplanted under the mammary pats of 35 mice. When the transplanted tumor volume had reached ≈100 mm^3^, the mice were assigned to seven groups randomly: Control, Free GMP, PTX Injection, P/G-Free, GMP NPs, PTX NPs, and P/G-NPs. Formulations were administered to the mice once every two days by three injections (iv) with a GMP dose of 16 mg/kg and PTX dose of 2.0 mg/kg. Mice in the control group were administered physiological (0.9%) saline only. The tumor volume and body weight of the mice were recorded once every two days. The tumor volume was calculated using Formula (3):

V = (W^2^ × L)/2, (3)

where V is the tumor volume, W is the smaller perpendicular diameter, and L is the larger perpendicular diameter. Tumor growth was normalized to the original volume calculated on the first day of measurement. The body weight of the mice in each group was documented. The mice were sacrificed two days after the final injection by CO_2_ asphyxiation, and the tumors were collected. One portion of the tumor was fixed in 10% formalin, paraffin-embedded, and sectioned for the terminal deoxynucleotidyl transferase-mediated nick end labeling (TUNEL) assay (Promega, Madison, WI, USA) and staining with hematoxylin and eosin (H&E). One portion of the tumor was stored at −80 °C for Western blotting.

### 4.10. Tissue Distribution

For the biodistribution study of nanoparticles in vivo, DiD-c-NPs were used for fluorescence detection. Tumor-bearing mice were injected with DiD-c-NPs (0.8 mg/kg) through the tail vein. Three mice were sacrificed, and their hearts, livers, spleens, lungs, kidneys, and tumors were collected after 24 h. For imaging and quantification, the organs and tumors were washed, weighed, and subjected to an IVIS kinetics optical system (PerkinElmer, CA, USA).

### 4.11. Tissue Analysis

Apoptotic and non-apoptotic cells in tumor tissues were evaluated by histology with H&E staining under the assistance of Dr. Shuliang Gao, a pathologist, at Jiangxi University of Traditional Chinese Medicine, and using TUNEL assay as recommended by the manufacturer (Promega, Madison, WI, USA). DAPI Mounting Medium was dropped on the sections for nuclear staining. TUNEL-positive (apoptotic) cells had pyknotic nuclei with dark-green fluorescent staining. Images of the sections were taken by a fluorescence inverted microscope (IX71; Olympus, Tokyo, Japan). The apoptotic index was calculated by dividing the number of TUNEL-positive cells by the total number of cells in the field. Ten randomly selected microscopic fields were analyzed quantitatively by ImageJ software (US National Institutes of Health). Western blotting was carried out on lysates generated from single-cell suspensions from tumors after different treatments, as described previously. Primary antibodies were directed against Bcl-2, Bcl-xL, Cleaved PARP, CRT, and tubulin (Abcam, Cambridge, MA, USA). After washing, the blots were incubated with horseradish peroxidase (HRP)-conjugated secondary antibodies (Abcam, Cambridge, MA, USA). Signals were detected using the Immobilon Western Chemiluminescent HRP Substrate (MilliporeSigma, Saint Louis, MO, USA). Western blotting was done in triplicate for each group, and the optical density of each protein band was analyzed by Quantity One software (Bio Rad Laboratories, Hercules, CA, USA).

### 4.12. Toxicity Analyses In Vivo

After three injections, blood was collected from the venous plexus of the eye and centrifuged at 4000 rpm for 5 min at room temperature. The organs (heart, liver, spleen, lung, and kidney) were fixed and sectioned for H&E staining, and examined under the assistance of Dr. Shuliang Gao, a pathologist, at Jiangxi University of Traditional Chinese Medicine. For the hematology assay, whole blood was also collected from healthy nude mice after the final injection. White blood cells (WBC), red blood cells (RBC), platelets (PLT), hematocrits (HCT), and hemoglobin (HGB) were calculated for the evaluation of myelosuppression. Serum levels of alanine aminotransferase (ALT), aspartate aminotransferase (AST), lactate dehydrogenase (LDH), creatinine, and blood urea nitrogen (BUN) were assayed as indicators of hepatic and renal function.

### 4.13. Statistical Analyses

All the results, except the in vivo data, are presented as the mean ± standard deviation (SD). The Student’s *t*-test and one-way analysis of variance were used to evaluate significance, and *p* < 0.05 was considered significant.

## 5. Conclusions

In conclusion, an effective and stable nanosystem with a long-circulating property and active targeting ability was developed, which exhibited high EE% and DL% of PTX and GMP. P/G-NPs acts significantly in inhibiting breast cancer growth, outperforming the delivery of free GMP and PTX injections with better tolerance.

## Figures and Tables

**Table 1:** Stability of P/G-NPs over 15 days.

	Particle size (nm)	Zeta Potential (mV)	EE ^1^ of GMP ^2^ (%)	EE of PTX ^3^(%)	DL ^4^ of GMP (wt % ^5^)	DL of PTX (wt %)
Day 1	85.1 ± 8.1	18.3 ± 0.63	93.6 ± 1.2	98.7 ± 0.5	6.3 ± 0.1	0.8 ± 0.004
Day 15	86.8 ± 6.9	17.4 ± 0.88	95.8 ± 3.8	97.5 ± 0.6	6.4 ± 0.3	0.8 ± 0.005

^1^ Entrapment efficiency. ^2^ Gemcitabine monophosphate. ^3^ Paclitaxel. ^4^ Drug loading. ^5^ percent by weight.

**Table 2:** Pharmacokinetic parameters of PTX and GMP in P/G-Free and P/G-NPs at a dose of 16 mg/kg for GMP and 2.0 mg/kg for PTX (*n* = 6).

	P/G-Free		P/G-NPs	
	PTX	GMP	PTX	GMP
AUC_0→ t_ (μg·mL^−1^·min^−1^)	114.53 ± 13.53	2265.97 ± 293.34	684.97 ± 99.93 *	10,324.06 ± 1801.59 ^§^
MRT_0→ t_ (min)	59.25 ± 4.13	92.45 ± 11.25	197.28 ± 17.08 *	210.68 ± 16.32 ^§^
CL^1^ (mL·min^−1^)	0.017 ± 0.003	0.0070 ± 0.0023	0.0028 ± 0.0011 *	0.0015 ± 0. 0004 ^§^
V_z_^2^ (L/kg)	1.49 ± 0.31	1.22 ± 0.35	0.74 ± 0.23 *	0.34 ± 0.11 ^§^
C_max_ (μg·mL^−1^)	5.10 ± 1.76	93.38 ± 18.87	17.04 ± 4.22 *	140.80 ± 25.22 ^§^

^1^ The total plasma clearance. ^2^ The apparent volume of distribution. * *p* < 0.05 vs. PTX in P/G-Free; ^§^ *p* < 0.05 vs. GMP in P/G-Free.

**Table 3:** Serum levels of AST, ALT, LDH, creatinine, and BUN after three injections in mice (*n* = 5).

	AST ^1^ (U/L)	ALT ^2^ (U/L)	LDH ^3^ (U/L)	Creatinine (mg/dL)	BUN ^4^ (mmol/L)
Control	103.3 ± 49.2	79.0 ± 6.4	95.3 ± 30.5	0.2 ± 0.2	8.4 ± 1.4
P/G-Free	178.3 ± 51.6	110.2 ± 21.7	100.1 ± 28.9	0.5 ± 0.1	10.3 ± 2.9
P/G-NPs	206.6 ± 38.4	98.6 ± 9.4	120.7 ± 41.2	0.4 ± 0.3	15.9 ± 2.1

^1^ Aspartate aminotransferase. ^2^ Alanine aminotransferase. ^3^ Lactate dehydrogenase. ^4^ blood urea nitrogen.
